# Synchronous primary glomus tumor in a patient with adenocarcinoma of the ipsilateral lung

**DOI:** 10.1111/1759-7714.13067

**Published:** 2019-04-05

**Authors:** Ju Sik Yun, Sang Yun Song, Kook Joo Na, Seok Kim, Yoo Duk Choi

**Affiliations:** ^1^ Department of Thoracic and Cardiovascular Surgery, Chonnam National University Hwasun Hospital Chonnam National University School of Medicine Jeollanam‐do South Korea; ^2^ Department of Pathology, Chonnam National University Hospital Chonnam National University School of Medicine Gwangju South Korea

**Keywords:** Glomus tumor, pathology, surgery

## Abstract

Glomus tumors are rare mesenchymal neoplasms arising from the glomus bodies in the deep dermis of the extremities or derive from the modified smooth muscle cells of the normal glomus body. Primary pulmonary glomus tumors are particularly rare and infrequently reported. We report a case of a primary glomus tumor occurring in the lung with adenocarcinoma in the ipsilateral lung as synchronous lung cancers in a 69‐year‐old man. He underwent lobectomy for adenocarcinoma and wedge resection for the glomus tumor with mediastinal lymph node dissection and was doing well without recurrence or metastasis at the last follow‐up.

## Introduction

Glomus tumors are rare mesenchymal neoplasms arising from the glomus bodies in the deep dermis of the extremities or derive from the modified smooth muscle cells of the normal glomus body. They account for less than 2% of soft tissue tumors^1^ and because of their rarity the exact incidence rate is unknown. Most glomus tumors are benign and occur mainly in the superficial soft tissues of the distal extremities, particularly the subungual regions of the hand, wrist, and foot.^2^ Cases of benign or malignant pulmonary glomus tumors have occasionally been reported. Herein, we report possibly the first case of a pulmonary glomus tumor that was simultaneously diagnosed with adenocarcinoma in the ipsilateral lung.

## Case report

**Figure 1:**
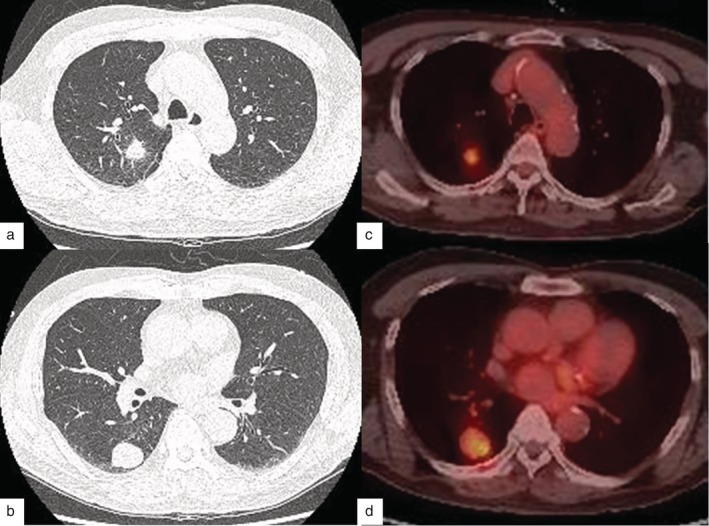
Chest computed tomography scans showed (**a**) an irregular nodular consolidation with mild spiculation and fissural retraction in the posterior segment of the right upper lobe (RUL) and (**b**) a well‐marginated mass in the superior segment of the right lower lobe (RLL). The positron emission tomography scan showed increased uptake (**c**) in the RUL (maximum standardized uptake value [SUVmax] 4.6) and (**d**) RLL (SUVmax 5.9).

A 69‐year‐old man presented to our hospital with lung nodules at the right upper and right lower lobes, which were detected at a different institution during an examination for intermittent hemoptysis. The patient's past medical history was significant for hypertension, with a smoking history of 50 pack‐years. Physical examination and routine laboratory tests revealed no significant findings. A chest computed tomography (CT) scan revealed a 2.1 × 1.7 cm sized mass in the posterior segment of the right upper lobe (RUL), along with a 3 × 2.3 cm sized mass in the superior segment of the right lower lobe (RLL)(Fig 1a). The nodule in the RUL was suspected to be primary lung cancer, while the nodule in the RLL was suspected to be a benign neoplasm or double primary lung cancer. Therefore, we performed a staging workup for suspected lung cancer. Positron emission tomography showed increased uptake only in the RUL and RLL (Fig 1b). Bronchoscopy showed no endobronchial involvement. As the pulmonary function test indicated no contraindication, we recommended surgery because of the possibility of malignancy in each nodule. Thoracoscopic wedge resection was performed with a frozen section for each nodule. The intraoperative frozen section revealed adenocarcinoma with poor differentiation in the RUL and non‐small cell lung cancer with very poor differentiation in the RLL. CT findings showed a RUL lesion with spiculated margins and a RLL lesion with well‐defined margins. We performed an additional upper lobectomy with mediastinal lymph node dissection after the thoracotomy considering the CT findings and an insufficient wedge resection margin of the RUL. The patient was discharged from the hospital without any complications on postoperative day eight.

**Figure 2:**
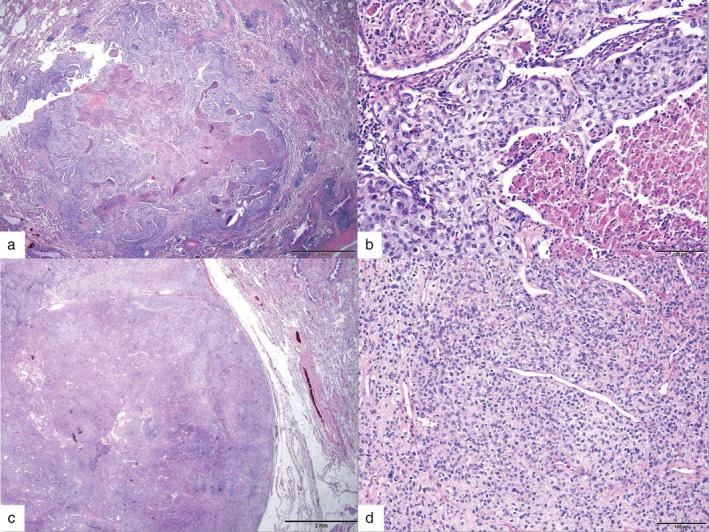
Histopathologic findings (hematoxylin & eosin staining). Right upper lobe mass: (**a**) the tumor showed central necrosis (x20) and (**b**) a solid growth pattern of tumor cells with atypical features (x200). Right lower lobe mass: (**c**) the tumor showed a well‐demarcated border (x20) and (**d**) monotonous epithelioid features of tumor cells with nuclear atypia (x100).

**Figure 3:**
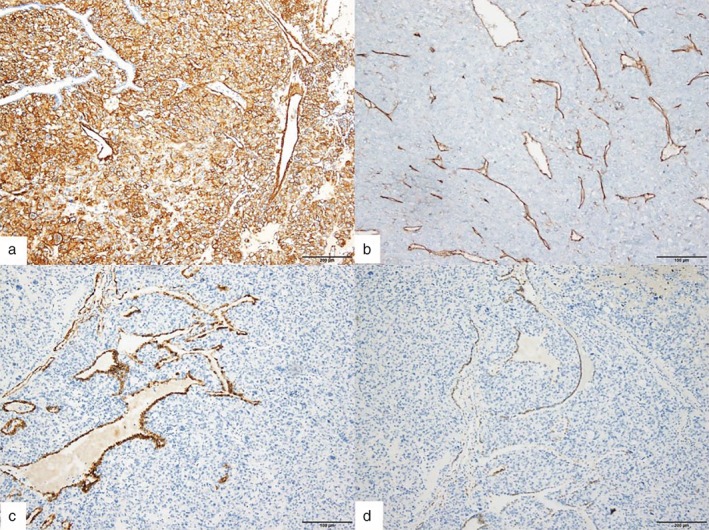
Immunohistochemistry staining of the right lower lobe mass. The tumor cells were (**a**) positive for actin and negative for (**b**) CD34, (**c**) cytokeratin, and (**d**) TTF‐1.

The results of the biopsy revealed that despite poor differentiation of the tumor cells, the RUL mass was an adenocarcinoma positive for CK7 and TTF‐1 (Fig 2a,b). The RLL mass was a tumor consisting mostly of cells that morphologically looked like epithelial cells, many of which showed apparent dysplasia and cell division (Fig 2c,d). This tumor was negative for CK, CD34, and TTF‐1, but positive for vimentin and actin (Fig 3). Based on these pathological findings, the final diagnosis was an adenocarcinoma in the RUL and a malignant glomus tumor in the RLL. As lymph node metastasis had not developed, no adjuvant chemotherapy or radiation therapy was administered. The patient has remained healthy and without recurrence for three years following the procedure and is currently in follow‐up at an outpatient setting.

## Discussion

This case is a rare instance where a pulmonary glomus tumor was simultaneously diagnosed with another primary adenocarcinoma of a different histologic type. Although most glomus tumors occur mainly in the superficial soft tissues, in rare cases, glomus tumors can form in deep soft tissue or visceral organs where glomus bodies are sparse or absent, such as in the gastrointestinal tract, central nervous system, bones, lungs, and mediastinum.^3^, ^4^, ^5^ Ariizumi *et al.* presented the clinicopathological features of 33 cases, including their own case that occurred in the respiratory tract.^6^ Overall, glomus tumors showed no gender predilection, but subungual lesions were more prevalent in women, while respiratory tract lesions were more prevalent in men. Approximately one‐third of the cases occurred in the bronchi and two‐thirds in the lungs. After excluding seven cases of glomangiosarcoma, the final diagnosis in all cases was a typical glomus tumor. Patients were either asymptomatic or complained of respiratory symptoms. The glomus body is thought to be associated with regulation of body temperature, but there were no symptoms related to this function. However, Huang *et al.* reported a right pulmonary glomus tumor with hyperpyrexia and anemia, which improved after thoracoscopic resection of the RUL, highlighting the association between the glomus tumor and hyperpyrexia.^7^

Histologically, glomus tumors can be classified as typical (solid glomus tumors, glomangiomas, and glomangiomyomas) or atypical, although most glomus tumors are from the former category. Folpe *et al.* analyzed and reported 52 cases that contained diagnoses of atypical or malignant glomus tumors.^8^ Atypical glomus tumors are classified into four subtypes: malignant glomus tumors, symplastic glomus tumors, glomangiomatosis, and glomus tumors of uncertain malignant potential. Each subtype was presented with corresponding criteria, and these subtypes were later added to the World Health Organization (WHO) classification of tumors of soft tissue and bone under the histopathology of glomus tumors.^9^ The criteria for diagnosis of malignant glomus tumors are: size > 2 cm, subfascial or visceral location, atypical mitotic figures or marked nuclear atypia, and any level of mitotic activity. Based on these criteria, we appropriately determined the tumor in this case to be a malignant glomus tumor.

Pulmonary glomus tumors must be distinguished from hemangiopericytomas, sclerosing hemangiomas, carcinoid tumors, and smooth muscle tumors such as leiomyomas.^10^ For a precise diagnosis, a detailed histopathologic examination must be accompanied by immunohistochemistry. Glomus cells are small, uniform, and round with a centrally placed, round nucleus. Each cell is surrounded by basal lamina. Occasionally, glomus cells show oncolytic or epithelioid changes. In immunohistochemical examination, glomus cells are typically positive for smooth muscle actin, pericellular type IV collagen, and H‐caldesmon. They are negative for desmin, CD34, CK, and S100 protein.^9^

Because of their rarity, the prognosis and clinical course of extracutaneous glomus tumors has not been well established. Most glomus tumors show a benign pattern of behavior, but they may also show aggressive and/or metastatic potential.^11^ Most authors recommend surgical resection for definitive histologic diagnosis and treatment.^12^ Surgical procedures should include implementation of radical resections with sufficient resection margins considering the malignant potential of these tumors. For intrapulmonary lesions, a sublobar resection is sufficient, but for large masses and those centrally located, lobectomy may be warranted.^13^

## Disclosure

No authors report any conflict of interest.
